# Characteristics associated with HIV and hepatitis C seroprevalence among sexual and injecting partners of HIV positive persons who inject drugs in Nairobi and coastal Kenya

**DOI:** 10.1186/s12879-022-07036-8

**Published:** 2022-01-21

**Authors:** Betsy C. Sambai, Hanley Kingston, Aliza Monroe-Wise, Loice Mbogo, Emily Juma, Natasha Ludwig-Barron, Brandon L. Guthrie, David Bukusi, Bhavna H. Chohan, John Scott, Rose Bosire, Matthew Dunbar, Paul Macharia, Sarah Masyuko, William Sinkele, Joshua T. Herbeck, Carey Farquhar

**Affiliations:** 1 HTC and HIV Care, Kenyatta National Hospital, Box 20723-00202, Nairobi, Kenya; 2 Institute of Public Health Genetics, University of Washington, UW Box #351620, Seattle, WA 98195 USA; 3 Department of Global Health, School of Public Health, University of Washington, UW Box #351620, Seattle, WA 98195 USA; 4 Department of Epidemiology, School of Public Health, University of Washington, UW Box #351619, Seattle, WA 98195 USA; 5 Centre for Clinical Research, Kenya Medical Research Institute (KEMRI), Box 54840-00200, Nairobi, Kenya; 6 Department of Medicine, Division of Allergy and Infectious Diseases, University of Washington, Box #356423, Seattle, WA 98195 USA; 7 Center for Studies in Demography and Ecology, University of Washington, Seattle, WA 98195 USA; 8 Support for Addictions Prevention and Treatment in Africa (SAPTA), Box #21761-00505, Nairobi, Kenya; 9 Global Assistance Program-Kenya, University of Washington, Box #20723-0020, Nairobi, Kenya; 10 National AIDS and STI Control Program, Ministry of Health, Box #13131-00202, Nairobi, Kenya; 11 Center for Virus Research, Kenya Medical Research Institute (KEMRI), Box 54840-00200, Nairobi, Kenya

**Keywords:** Persons who inject drugs (PWID), HIV, Hepatitis C (HCV), Sub-Saharan Africa (SSA)

## Abstract

**Background:**

Persons who inject drugs (PWID) have higher HIV and hepatitis C virus (HCV) seroprevalence than the general population in many parts of sub-Saharan Africa (SSA). The seroprevalences of HIV and HCV are also higher in coastal Kenya than in Nairobi. Understanding drivers of regional HIV and HCV variation among PWID in Kenya may inform population-specific prevention interventions.

**Methods:**

Using a cross-sectional study, we defined HIV and HCV seroprevalence among persons identified as sexual or injecting partners of HIV positive PWID in two regions of Kenya and used logistic regression to identify demographic and behavioral characteristics associated with higher seroprevalence.

**Results:**

Among 2386 partners, 469 (19.7%) tested HIV positive and 297(12.4%) tested HCV antibody positive. Partners on the Coast were more likely to live with HIV (seroprevalences: Coast = 23.8%, Nairobi = 17.1%; p < 0.001) and be HCV antibody positive (seroprevalences: Coast = 17.0%, Nairobi = 8.6%; p < 0.001). After adjusting for sex, age, and years injecting and accounting for clustering by site, the higher prevalence of both diseases in the Coast remained significant for HIV (OR 1.68, 95% CI 1.13–2.51) but not for HCV (OR 1.72, 95% CI 0.84–3.74). Compared to those recruited in Nairobi, partners on the Coast were older (Coast = 35 years, Nairobi = 31 years; p < 0.001), more likely to be male (Coast = 77.6%, Nairobi = 61.7%; p < 0.001), to have paid (Coast = 59.2%, Nairobi = 32.8%; p < 0.001) or received (Coast = 44.2%, Nairobi 35.4%; p < 0.001) money for sex, or to have had sex with someone they knew to be HIV positive (Coast 22.0%, Nairobi 10.8%; p < 0.001). Partners who had injected for five or more years had 1.48 times greater odds (95% CI 1.20–1.82) of living with HIV compared to partners who injected less than 5 years and more than twice the odds of HCV (95% CI 1.84–4.11).

**Conclusion:**

HIV and HCV seroprevalence among sexual and injecting partners of PWID was, respectively, 5 times and > 12 times greater than is reported among the general population in Kenya (4% and < 1%, respectively). Providing resources and education will be crucial to reduce exposure and to maintain the lower needle and equipment sharing that we observed compared to other studies.

**Supplementary Information:**

The online version contains supplementary material available at 10.1186/s12879-022-07036-8.

## Background

Globally, an estimated 37.7 million people were living with HIV in 2020, and the World Health Organization estimates there were approximately 58 million people living with chronic hepatitis C (HCV) in 2019 [1]. Of the estimated 15.6 million people aged 15–64 years who inject drugs, about 17.8% and 52.3% (based on antibody positivity) live with HIV and HCV, respectively [2]. In the 1990s, tourism and proximity to drug shipment routes introduced substantially higher levels of opiates and cocaine to the coastal regions of Africa, precipitating increasing rates of injection drug use (IDU) [3] and injecting behaviours associated with increased risk of HIV [4]. The World Health Organization estimates that in sub-Saharan Africa, people who inject drugs (PWID) perform about 18% of injections with reused syringes or unsterilized needles [5], placing them at risk of acquiring blood borne infections like HIV and HCV. In Kenya, heroin use is primarily restricted to urban areas, particularly coastal cities like Malindi and Mombasa, although drug trafficking routes are spreading inland to cities like Nairobi [6]. In response to increasing levels of IDU, the Kenyan Government introduced needle and syringe programs (NSP) in 2013 and methadone maintenance treatment in 2014 [7, 8]. Most PWID in Africa are male, with injecting drug use among females overrepresented among those who receive money for sex [3].

SSA accounts for approximately 70% of global HIV infections [9], and Kenya accounts for 6% of the global cases, but 7% of new HIV infections [10]. Thirty-three percent of new infections in Kenya occur in key populations: PWID, sex workers, and men who have sex with men [11]. Estimates of the prevalence of HIV among PWID in SSA vary widely from 6 to 43% between different countries [12], and in Kenya it is estimated to be 18% (20.5% in the coastal region and 14.5% in Nairobi), 3-times higher than in the general population [13, 14]. While approximately 7.5% of new HIV infections in Kenya are thought to result from IDU, this figure is much higher (18.7%) on the Coast [13].

Three to four million new HCV infections occur globally each year, and most people living with HCV are unaware of their infection [5, 15]. Estimates for the prevalence of HCV in SSA range from 3.0 to 5.3% [6, 16]. Although this prevalence is estimated to be lower in Kenya (approximately 0.9%) [5, 16], these numbers published in 2002, don’t reflect the more recent negative effects of increasing levels of IDU in the country or positive effects of interventions like needle-syringe programs and methadone clinics. Estimates of HCV seroprevalence among PWID in Kenya differ substantially, with a small 2005 study showing a 61% prevalence of HCV among 101 people who inject heroin [17], but a larger 2019 study finding only 13% of PWID to be living with HCV [6]. No studies that we are aware of looked at the prevalence of IDU among the sexual and injecting partners of PWID. In Kenya, a higher HCV seroprevalence has been observed among PWID in coastal Kenya (22%) compared to Nairobi (13%), with low seroprevalence in Western Kenya (1%) [6], but little is known about what drives this difference.

Additionally, no research that we know of has looked at HIV and HCV prevalence among sexual and injection partners of PWID in Kenya and sub-Saharan Africa. A study among female partners of male persons who injected drugs in Iran documented a higher HIV (7.7% vs 2.8%) and HCV (36.6% vs 8.4%) prevalence among female partners who injected drugs compared to non-injecting drug users [18]. These prevalences were lower than that of the male PWID. The same trend was observed among female partners of male injecting drug users in Kazakhstan [19]. Determining the prevalence of and risk factors for HIV and HCV among partners, identified through assisted partner services (APS), in Kenya is critical to identifying and reaching people with or at risk of contracting these diseases, especially given increasing numbers of PWID [20] and drastically improved treatment options for both diseases within the last decade. In this paper, we identified demographic and behavioral characteristics associated with HIV and HCV seroprevalence among the sexual and injecting partners of PWID living with HIV in coastal Kenya and Nairobi, two regions with high levels of IDU and HIV [6, 13, 14], with the goal of informing tailored interventions in Kenya and other parts of SSA.

## Methods

### Study design

This is a cross-sectional study nested in the Study of HIV, HCV, APS, and Phylogenetics for PWID (SHARP), a prospective cohort study that recruited participants from 2018 to 2020 and used APS to identify and test the sexual and injecting partners of HIV positive PWID. APS involves collecting partner contact information from persons testing positive for HIV and using health advisors to offer testing and referrals after notification of exposure. HIV and HCV seroprevalence among partners was determined and compared between the coastal and Nairobi regions.

### Study participants and sites

A total of 768 HIV positive PWID (indexes) and 2,462 sexual and injecting partners were recruited from Nairobi (central Kenya) and Kilifi and Mombasa counties (coastal region) using convenience sampling. In Nairobi, we recruited participants from the two methadone sites at the Drug Rehabilitation Unit in Mathari Hospital and Ngara Health Center and three NSP sites managed by a harm reduction organization, the Support for Africa Addiction Prevention Treatment in Africa (SAPTA). At the coast region, we recruited from one methadone clinic at Malindi County Hospital and four NSP sites including Reachout program in Mombasa, Muslim Education Welfare Association (MEWA) sites in Mtwapa and Kilifi, and the Omari Project in Malindi. Index participants were enrolled if they were ≥ 18 years old, injected at least once in the past year, tested positive for HIV, gave locator information of their sexual or injecting partners and provided written informed consent for participation. Participation was considered a risk for index participants who had experienced intimate partner violence in the last 1 month, so they were excluded. We did not analyze data from indexes in this paper. Partners who were ≥ 18 years, had sexual intercourse and/or injected with the index participant in the past three years and gave consent to participate in the study were eligible for inclusion in our study.

### Study procedures

The study procedures are reported in the published study protocol [21]. In summary, individuals who were known to have HIV or who tested positive for HIV at the study sites were invited to enroll as indexes into the study. The study health advisors obtained information (names, telephone contacts, and residence) about each index’s sexual and injecting partners.

Partners to the index case were contacted either by phone or through physical tracing by peer educators guided by the study health advisor while keeping the identity of the index anonymous. Once successfully traced, partners were invited to enroll in the study.

Socio-demographic data, HIV and hepatitis history, and sexual and drug use history was obtained for all participants. Rapid HIV testing using fingerstick samples was performed for partners during the interview sessions following the Kenya national algorithm [22] and HCV antibody testing was performed using the Abbott SD Bioline rapid one-step HCV testing kit (Abbott Pharmaceuticals, Chicago, IL) [23].

Data was collected using questionnaires programmed into tablets using Open Data Kit (ODK). All the data collected was uploaded to Ministry of Health National AIDS and STI Control Program (NASCOP) servers over an encrypted connection. Participants were compensated for travel expenses.

### Statistical analysis

We pre-selected twenty-eight socio demographic and behavioral characteristics to analyze. The choice of variables reflects our hypotheses, based on prior literature, for factors that could be associated with HIV and HCV seroprevalence and might explain regional differences, such as sexual and injecting behaviors and prior testing and results [6, 13, 24]. Continuous variables (age and number of times injecting each month) were described using median, inter-quartile range (IQR), means, and standard deviation (SD). The remaining variables were treated as categorical and described as count and proportions. We assessed all variables overall and stratified by region (Nairobi and Coast), and we used Fisher’s exact test (categorical variables) and t-test (continuous variables) to test for regional differences in the distributions of partner characteristics. We analyzed only baseline data (from the first enrollment), except to define partner type which reflected whether the person was named as sexual or injecting partners or as both types of partners any time they were enrolled. The distribution of partner characteristics across the study and by region is reported in Table 1.

**Table 1:** Distribution of partner characteristics by region

	Coast (N = 1026)	Nairobi (N = 1360)	Total (N = 2386)	p value*
Sociodemographic characteristics				
Partner type				< 0.001
Both sexual and injecting	236 (23.4%)	211 (15.6%)	447 (19.0%)	
Injecting	602 (59.7%)	928 (68.7%)	1530 (64.9%)	
Sexual	170 (16.9%)	211 (15.6%)	381 (16.2%)	
Identified by multiple partners	290 (28.3%)	192 (14.1%)	482 (20.2%)	< 0.001
Male	796 (77.6%)	839 (61.7%)	1635 (68.5%)	< 0.001
Age				< 0.001
Mean (SD)	35.3 (7.6)	31.8 (8.0)	33.3 (8.0)	
Median (IQR)	35 (30, 41)	31 (25, 37)	33 (27, 39)	
Marital status				< 0.001
Single	319 (31.1%)	682 (50.1%)	1001 (42.0%)	
Divorced	268 (26.1%)	276 (20.3%)	544 (22.8%)	
Partnered	93 (9.1%)	53 (3.9%)	146 (6.1%)	
Married or widowed	346 (33.7%)	349 (25.7%)	695 (29.1%)	
Have stable housing	915 (89.2%)	1150 (84.6%)	2065 (86.5%)	0.0011
Experienced physical violence (past year)	452 (44.1%)	416 (30.6%)	868 (36.4%)	< 0.001
HIV/HCV history and test results				
Previously tested for HIV	928 (90.4%)	1328 (97.6%)	2256 (94.6%)	< 0.001
Previously tested positive for HIV	212 (20.7%)	194 (14.3%)	406 (17.0%)	< 0.001
Previously tested for HCV	128 (12.5%)	374 (27.5%)	502 (21.0%)	< 0.001
Previously tested seropositive for HCV	35 (3.4%)	45 (3.3%)	80 (3.4%)	0.91
HIV positive test (95% CI)	239 (23.3%) (20.7–26.0)	230 (16.9%) (15.0–19.0)	469 (19.7%) (18.1–21.3)	< 0.001
HCV seropositive test (95% CI)	179 (17.4%) (15.1–19.9)	118 (8.7%) (7.2–10.3)	297 (12.4%) (11.1–13.8)	< 0.001
Sexual history				
Number of sexual partners (past 3 months)				< 0.001
0	330 (32.2%)	811 (59.7%)	1141 (47.9%)	
1–2	483 (47.1%)	390 (28.7%)	873 (36.6%)	
> 2	212 (20.7%)	157 (11.6%)	369 (15.5%)	
Received money for sex (ever)	453 (44.2%)	481 (35.4%)	934 (39.2%)	< 0.001
Gave money for sex (ever)	607 (59.2%)	446 (32.8%)	1053 (44.2%)	< 0.001
Had sex with someone knew to be HIV positive (ever)	226 (22.0%)	147 (10.8%)	373 (15.7%)	< 0.001
Used a condom when last had sex	440 (43.6%)	633 (46.9%)	1073 (45.5%)	0.11
DRUG USE				
Used heroin (past month)	877 (85.5%)	1286 (94.6%)	2163 (90.7%)	< 0.001
Used benzos (past month)	170 (16.6%)	246 (18.1%)	416 (17.4%)	0.35
Used cocaine (past month)	113 (11.0%)	69 (5.1%)	182 (7.6%)	< 0.001
Used alcohol (past month)	347 (33.8%)	443 (32.6%)	790 (33.1%)	0.54
Years injecting				0.085
0 (don't inject)	95 (9.3%)	121 (8.9%)	216 (9.1%)	
< 5	540 (52.6%)	776 (57.1%)	1316 (55.2%)	
≥ 5	391 (38.1%)	463 (34.0%)	854 (35.8%)	
Injecting behaviors**				
Times injecting per month				0.49
Mean (SD)	70.1 (51.5)	72.4 (94.0)	71.4 (78.6)	
Median (IQR)	60 (30, 90)	60 (56, 90)	60 (30, 90)	
Shared needles (past month)	22 (2.4%)	72 (5.8%)	94 (4.3%)	< 0.001
Shared equipment (past month)	27 (2.9%)	187 (15.1%)	214 (9.9%)	< 0.001
Injected blood (past month)	14 (1.5%)	18 (1.5%)	32 (1.5%)	1
On methadone now	331 (35.6%)	146 (11.8%)	477 (22.0%)	< 0.001

p values are from a test for a significant difference in distribution of partner characteristic by region

^a^For categorical variables: Fisher’s exact test (with simulated p-values for 200-replicates when > 2 categories), for continuous variables: t-test

^b^Variables in this group are analyzed only among people who report having injected drugs

We used separate logistic regression models (reporting 95% confidence intervals) to test the association between each partner characteristic (independent variables) and HIV (Table 2) or HCV (Table 3) seroprevalence (dependent variables), both overall and stratified by region. Recruitment site was included as a clustering effect and sex, age, and years injecting (categorical) were included as adjustment variables based on prior literature and domain knowledge suggesting these could be strongly associated with HIV/HCV seroprevalence and/or regional differences in that seroprevalence. The same adjustment variables were included for each test presented in Tables 2 and 3, but secondary analyses were performed to better understand the role of other variables in some of the associations. For example, we tested the association between condom use and HIV positivity only among individuals without a prior positive test and investigated sex differences in sexual behaviors and in HCV positivity among PWID. We also tested for multiplicative interaction by region in the effects of each characteristic on HIV and HCV positivity. Where interaction was observed with statistical support p < 0.01, we report only the OR stratified by region, as the combined OR is not considered to be informative [25]; however, the combined OR should also be interpreted with caution for variables where there is modest evidence of interaction by region. The non-stratified test and interaction test additionally included region as an adjustment variable. Analyses were conducted using R statistical software [26].

**Table 2:** Associations between partner characteristics and living with HIV

Strata (reference group = No, unless otherwise indicated)		OR (CI 95%)								p (interaction by region)
		Total		Total		Coast		Nairobi		
		Adjusted for sex and age, and years injecting		Adjusted for sex, age, years injecting, and region		Adjusted for sex and age, and years injecting		Adjusted for sex, age, and years injecting		
Sociodemographic characteristics										
Enrolled as a sexual partner (ref = enrolled only as an injecting partner)**	2.18 (1.65–2.87)*		2.06 (1.58–2.69)*		2.12 (1.29–3.47)*		1.94 (1.56–2.40)*		0.67	
Enrolled as an injecting partner (ref = enrolled only as a sexual partner)**	0.84 (0.60–1.16)		0.86 (0.67–1.09)		0.81 (0.52–1.27)		0.92 (0.79–1.07)		0.87	
Identified by multiple partners	3.89 (2.76–5.47)*		3.66 (2.53–5.29)*		2.79 (1.54–5.04)*		4.88 (3.64–6.55)*		0.10	
Enrolled in Nairobi (ref = Coast)	1.68 (1.13–2.51)*									
Male	0.20 (0.16–0.25)*		0.18 (0.14–0.23)*		0.16 (0.10–0.25)*		0.20 (0.18–0.22)*		0.03*	
Age (× 10)					1.58 (1.26–1.98)*		2.62 (1.80–3.83)*		0.004*	
Marital status (ref = single)										
divorced**		0.95 (0.76–1.19)		0.90 (0.74–1.09)		0.85 (0.61–1.18)		0.94 (0.68–1.29)		0.04*
partnered**		1.10 (0.72–1.67)		0.90 (0.52–1.53)		0.98 (0.55–1.76)		0.51 (0.08–3.41)		0.47
married or widowed**		1.20 (1.03–1.41)*		1.12 (0.91–1.38)		1.05 (0.72–1.51)		1.14 (0.85–1.54)		0.33
Have stable housing		1.14 (0.87–1.48)		1.10 (0.84–1.44)		1.01 (0.74–1.36)		1.12 (0.71–1.77)		0.57
Experienced physical violence (past year)		1.19 (0.92–1.53)		1.09 (0.93–1.28)		1.10 (0.83–1.46)		1.01 (0.91–1.12)		0.32
HIV/HCV history and test results										
Previously tested for HIV		0.41 (0.19–0.90)*		0.48 (0.20–1.16)		0.60 (0.18–1.95)		0.27 (0.19–0.36)*		0.23
Previously tested for HCV		1.86 (0.95–3.64)		2.13 (1.28–3.56)*		3.52 (2.99–4.14)*		1.67 (0.78–3.56)		0.02*
HCV seropositive test		2.48 (1.75–3.53)*		2.33 (1.75–3.12)*		2.35 (1.63–3.38)*		2.42 (1.35–4.35)*		0.67
Sexual history										
Number of sexual partners (past 3 months) (ref = 0)										
1–2						1.34 (0.96–1.88)		0.90 (0.82–0.98)*		0.002*
> 2		1.42 (1.16–1.74)*		1.23 (1.01–1.50)*		1.27 (0.79–2.02)		1.34 (0.91–1.98)		0.48
Received money for sex (ever)		1.23 (0.74–2.03)		1.11 (0.63–1.95)		0.66 (0.55–0.80)*		2.02 (0.98–4.14)		0.06
Gave money for sex (ever)						0.67 (0.57–0.78)*		0.89 (0.76–1.04)		0.004*
Sex with someone Knew to be HIV Positive		3.96 (2.57–6.10)*		3.71 (2.31–5.96)*		3.04 (1.88–4.93)*		4.56 (2.27–9.16)*		0.35
Used a condom when last had sex		1.52 (1.26–1.84)*		1.55 (1.26–1.90)*		1.37 (0.93–2.03)		1.79 (1.54–2.09)*		0.16
Drug use										
Used heroin (past month)		0.71 (0.40–1.28)		0.82 (0.47–1.43)		0.70 (0.39–1.24)		1.03 (0.24–4.34)		0.7
Used benzos (past month)						0.92 (0.76–1.12)		0.61 (0.50–0.74)*		< 0.001*
Used cocaine (past month)		0.92 (0.68–1.25)		0.85 (0.57–1.24)		0.84 (0.59–1.20)		0.83 (0.30–2.26)		0.94
Used alcohol in past month		0.90 (0.66–1.23)		0.90 (0.69–1.16)		0.72 (0.53–0.98)*		1.03 (0.72–1.48)		0.12
Years injecting (ref = < 5 years)										
never injected		0.69 (0.41–1.15)		0.65 (0.33–1.26)		0.53 (0.17–1.61)		0.70 (0.43–1.16)		0.64
≥ 5 years		1.44 (1.22–1.71)*		1.48 (1.20–1.82)*		1.70 (1.34–2.16)*		1.30 (1.12–1.50)*		0.72
Injecting behaviors***										
Times injecting per month (X30)		1.03 (0.97–1.09)		1.03 (0.98–1.08)		0.92 (0.80–1.05)		1.07 (0.87–1.32)		0.15
Shared needles (past month)		1.17 (0.92–1.50)		1.32 (0.99–1.75)		1.81 (0.81–4.03)		1.14 (0.77–1.69)		0.20
Shared equipment (past month)		1.31 (0.96–1.79)		1.63 (1.34–1.99)*		1.01 (0.56–1.81)		1.76 (1.64–1.89)*		0.13
Injected blood (past month)		1.72 (0.92–3.24)		1.72 (0.84–3.52)		–		–		–
On methadone now		1.35 (0.83–2.20)		1.13 (0.73–1.75)		1.33 (0.86–2.07)		0.64 (0.32–1.27)		0.05

Where not otherwise indicated, the reference category is no or 0

– insufficient sample size

*Significant at alpha = 0.05

**Participants enrolled as sexual partners includes those who were named as both sexual and injecting partners as does participants enrolled as injecting partners

***Variables in this group are analyzed only among people who report having injected drugs

**Table 3:** Association between partner characteristics and testing seropositive for HCV antibodies

Strata (reference group = No, unless otherwise indicated)	OR (CI 95%)				p (interaction by region)
	Total	Total	Coast	Nairobi	
	Adjusted for sex and age, and years injecting	Adjusted for sex, age, years injecting, and region	Adjusted for sex and age, and years injecting	Adjusted for sex, age, and years injecting	
Sociodemographic characteristics					
Enrolled as a sexual partner (ref = enrolled only as an injecting partner)**	1.25 (0.86–1.81)	1.20 (0.88–1.64)	1.01 (0.70–1.45)	1.53 (0.80–2.93)	0.19
Enrolled as an injecting partner (ref = enrolled only as a sexual partner)**	1.38 (0.80–2.40)	1.36 (0.76–2.46)	2.23 (0.97–5.14)	0.85 (0.50–1.46)	0.04*
Identified by multiple partners	2.84 (1.54–5.26)*	2.57 (1.33–4.97)*	2.81 (1.01–7.81)*	2.24 (1.02–4.91)*	0.72
Enrolled on the Coast (ref = Nairobi)	1.72 (0.84–3.74)				
Male	1.88 (1.03–3.45)*	1.67 (0.99–2.80)	1.78 (1.02–3.09)*	1.61 (0.65–3.98)	0.79
Age (× 10)	1.34 (1.15–1.57)*	1.25 (1.07–1.45)*	1.22 (1.06–1.40)*	1.28 (0.92–1.79)	0.94
Marital status					
Divorced**	0.66 (0.44–0.99)*	0.61 (0.43–0.87)*	0.63 (0.48–0.83)*	0.50 (0.20–1.25)	0.43
Partnered**			0.32 (0.15–0.69)*	1.06 (0.55–2.01)	0.009*
Married or widowed**	0.78 (0.47–1.27)	0.71 (0.47–1.07)	0.55 (0.43–0.71)*	0.96 (0.45–2.04)	0.07
Have stable housing			1.13 (0.73–1.76)	0.55 (0.41–0.74)*	0.003*
Experienced physical violence (past year)			1.01 (0.89–1.15)	0.74 (0.59–0.93)*	0.005*
HIV/HCV history and test results					
Previously tested for HIV	1.35 (0.78–2.33)	1.73 (1.10–2.72)*	1.68 (1.05–2.68)*	2.15 (0.17–26.85)	0.84
Previously tested for HCV	2.24 (0.92–5.47)	2.71 (1.45–5.07)*	3.64 (2.69–4.93)*	2.12 (0.55–8.14)	0.4
Positive HIV test	2.48 (1.74–3.54)*	2.35 (1.74–3.18)*	2.36 (1.65–3.38)*	2.37 (1.32–4.27)*	0.91
Sexual history					
Number of sexual partners (past 3 months) (ref = 0)					
1–2	0.94 (0.66–1.35)	0.76 (0.54–1.08)	0.76 (0.44–1.31)	0.72 (0.44–1.19)	0.87
> 2	0.77 (0.51–1.17)	0.57 (0.36–0.92)*	0.43 (0.23–0.78)*	1.06 (0.90–1.25)	0.01*
Received money for sex (ever)			0.58 (0.44–0.77)*	0.91 (0.81–1.01)	0.004*
Gave money for sex (ever)	1.02 (0.72–1.43)	0.88 (0.57–1.37)	0.77 (0.65–0.91)*	1.05 (0.40–2.74)	0.49
Sex with someone knew to be HIV positive	1.40 (1.03–1.89)*	1.23 (0.94–1.61)	1.14 (0.84–1.54)	1.43 (0.81–2.52)	0.52
Used a condom when last had sex	1.09 (0.94–1.26)	1.11 (0.97–1.28)	1.04 (0.84–1.29)	1.22 (0.99–1.50)	0.29
Drug use					
Used heroin (past month)			1.50 (1.07–2.12)*	0.51 (0.31–0.84)*	< 0.001*
Used benzos (past month)	1.09 (0.76–1.57)	1.12 (0.87–1.44)	1.02 (0.69–1.52)	1.26 (0.81–1.96)	0.5
Used cocaine (past month)	0.74 (0.41–1.33)	0.66 (0.39–1.12)	0.67 (0.41–1.11)	0.63 (0.10–3.98)	0.94
Used alcohol in past month			0.70 (0.58–0.84)*	–	–
Years injecting (ref = < 5 years)					
Never injected	–		–	–	0.71
≥ 5 years	2.62 (1.96–3.51)*	2.75 (1.84–4.11)*	3.11 (1.61–6.01)*	2.34 (1.69–3.24)*	0.48
Injecting behaviors***					
Times injecting per month (× 30)			1.20 (1.05–1.37)*	1.01 (0.99–1.03)	0.006*
Shared needles (past month)	1.03 (0.42–2.49)	1.16 (0.56–2.40)	0.99 (0.55–1.78)	1.26 (0.40–3.92)	0.68
Shared equipment (past month)	1.39 (0.65–2.97)	1.85 (0.99–3.45)	2.10 (0.77–5.74)	1.78 (0.79–4.02)	0.75
Injected blood (past month)	0.86 (0.22–3.37)	0.85 (0.19–3.76)	–	–	–
On methadone now	1.47 (0.96–2.25)	1.23 (0.79–1.92)	0.95 (0.57–1.58)	2.05 (1.26–3.35)*	0.029*

Where not otherwise indicated, the reference category is no or 0

– insufficient sample size

*Significant at alpha = 0.05

**Participants enrolled as sexual partners includes those who were named as both sexual and injecting partners as does participants enrolled as injecting partners

***Variables in this group are analyzed only among people who report having injected drugs

## Results

### Overall characteristics of the study population

The study enrolled 2,462 (Nairobi = 1,079, Coast = 1,383) partners of HIV positive PWID across 10 sites within Nairobi and coastal Kenya. Participants were excluded if they lacked data on age (n = 5), sex (n = 5), number of years injecting (n = 53), region recruited (n = 0), or HIV (n = 11) or HCV (n = 17) test result (Additional file 1: Table S1). The final dataset consisted of 2,386 participants (Nairobi = 1,026, Coast = 1,360) (Table 1, Additional file 1: Table S1 and Table S2).

Sixty-eight percent of participants were male, with a median age of 33 years [interquartile range (IQR): 27, 39]. The majority (64.9%) were identified only as an injecting partner, while 16.2% were identified only as a sexual partner, and 19.0% were identified as being both a sexual and injecting partner of either the same person or multiple people. The majority (91.0%) of participants reported having injected drugs, with a mean of 5.2 years injecting (SD: 4.9). Among participants who had injected drugs, 4.3% reported sharing needles and 9.9% reported sharing equipment in the last month.

The majority of partner participants had no sexual partner (47.9%) or 1–2 (36.6%) sexual partners in the last 3 months (Table 1). Of the 15.5% of participants reporting > 2 sexual partners in the last 3 months, 56.6% were female and 84.3% reported receiving money for sex. Persons who received money for sex were more than twice as likely to report having ever had sex with a person they knew to be HIV positive (23.7%) compared to those who had not (10.5%; p < 0.001) and were slightly more likely to report using a condom the last time they had sex (50.0% vs 42.7%; p = 0.002). HIV prevalence was greater than HCV prevalence, with 20.0% (CI 18.1–21.3) of participants testing seropositive for HIV and 12.2% (CI 11.1–13.8) for HCV.

### Regional differences in partner characteristics

Partner characteristics showed heterogeneity between the two regions (Table 1). Sexual behaviors associated in other studies with acquisition of sexually transmitted infections tended to be more common in the Coast, while several risk-associated injecting behaviors were more common in Nairobi. Participants on the Coast were older than in Nairobi (median age 35 vs 31 years, p < 0.001) and significantly more likely to be male (77.6% vs 61.7%). Participants on the Coast also reported more sexual partners in the previous three months and were also more likely to have paid or received money for sex; to have had sex with someone they knew to be HIV positive; and to be identified through an index who was a sexual partner. There was no significant regional difference in whether participants reported having used a condom the last time they had sex, but females who received money for sex were less likely to use a condom in the Coast (48.4%) compared to Nairobi (59.4%; p < 0.01). The majority of people who received money for sex in Nairobi were female (75.6%), while men made up the majority of those who received money for sex on the Coast (57.4%).

The percent of participants who reported ever having injected drugs (Coast = 90.7%, Nairobi = 91.1%), was very similar between both regions, but heroin use was higher in Nairobi (Table 1). Although overall levels were low, needle-sharing was twice as common (Coast = 2.4%, Nairobi = 5.8%; p < 0.001) and equipment sharing five-times as common (Coast = 2.9%, Nairobi = 15.1%; p < 0.001) in Nairobi. On the Coast, 23.8% (CI 20.7–26.0) of participants tested positive for HIV and 17.0% (CI 1.5–19.9) for HCV. In Nairobi, 17.1% (CI: 15.0–19.0) of participants tested positive for HIV and 8.6% (CI 7.2–10.3) for HCV (Table 1).

### Overall partner characteristics associated with HIV and HCV seropositivity

**Fig. 1:**
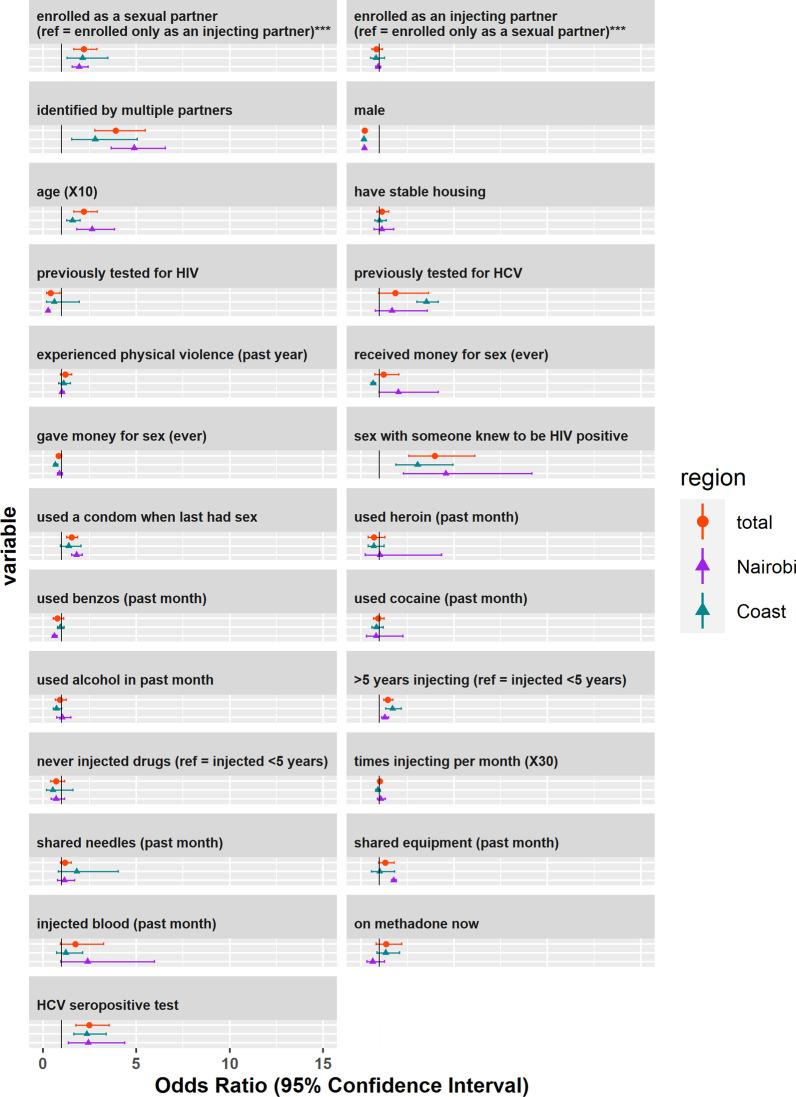
Associations between partner characteristics and living with HIV. *Note* associations by marital status are excluded

Several characteristics were associated with positive test results for both diseases in analyses controlling for sex, age and region. Individuals who were 10 years older had much higher odds (Coast: 95% CI 1.26–1.98; Nairobi: 95% CI 1.80–3.83) of living with HIV in both regions, while those injecting five or more years had 1.48 times greater odds (95% CI 1.20–1.82) of living with HIV compared to PWID who injected less than 5 years. Participants who shared equipment in the last month had 1.63 times higher odds (95% CI 1.34–1.99) of living with HIV. Participants identified by more than one index, those who were previously tested for HCV, and those testing HCV seropositive were significantly more likely to test positive for HIV (Table 2). Female participants were more likely to test HIV positive (OR 5.56, 95% CI 4.35–7.14) compared to male participants (Table 2, Fig. 1). Sexual behaviors significantly associated with a positive HIV test were: being identified through a sexual partner, having more than 2 sexual partners in the prior 3 months, giving money for sex, having used a condom the last time they had sex, and having had sex with someone they knew to be HIV positive. The negative association between condom use and HIV disappeared when restricted to persons who reported no prior knowledge of having HIV or HCV prior to enrolment.

**Fig. 2:**
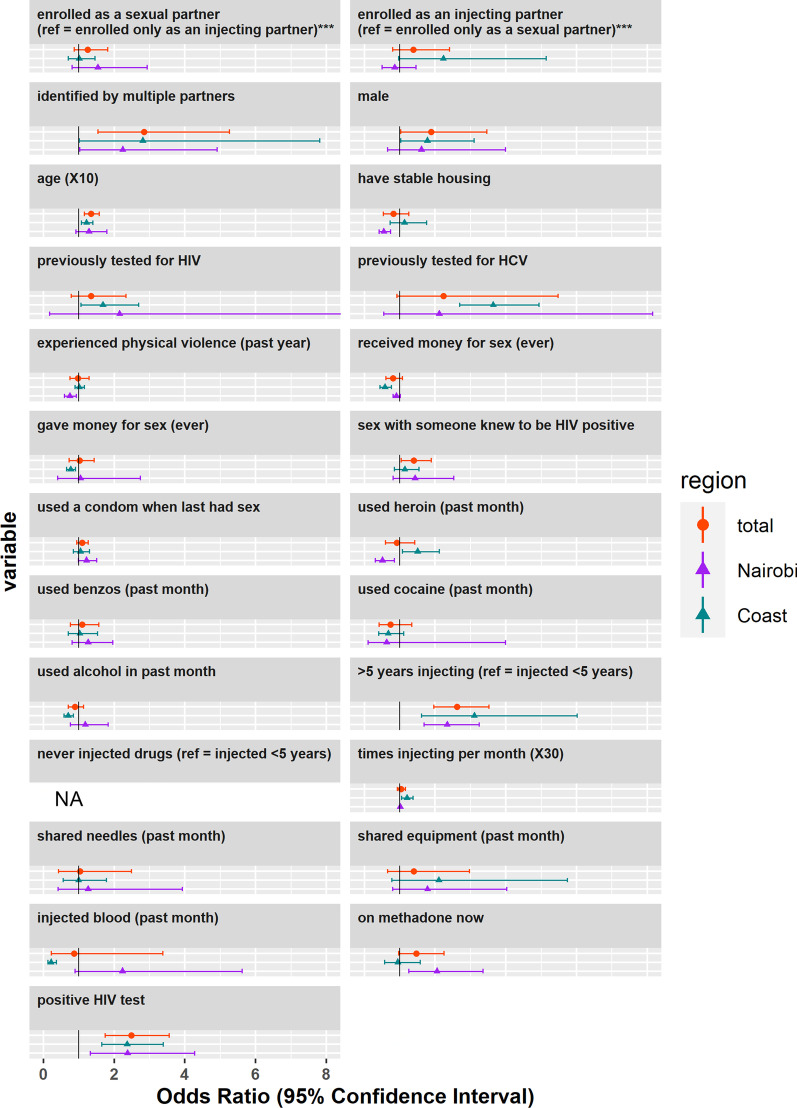
Associations between partner characteristics and living with HCV. *Note* associations by marital status are excluded

Participants who tested positive for HIV had 2.35 times (95% CI 1.74–3.18) higher odds of being HCV-antibody positive than HIV-negative participants. Individuals who were 10 years older were 1.25 times (95% CI 1.07–1.45) more likely to test HCV seropositive, while those injecting for five or more years had 2.75 times (95% CI 1.84–4.11) the odds of testing HCV seropositive. Participants who shared equipment in the last month had 1.85 times higher odds (95% CI 0.99–3.45) of testing HCV seropositive, although this effect was not significant. Participants identified by more than one index and those who were previously tested for HCV were significantly more likely to be seropositive for HCV (Table 3). Male participants were more likely to test HCV seropositive (OR 1.67, 95% CI 0.99–2.88) compared to female participants, although this result was not significant (Table 3, Fig. 2). Other characteristics associated with higher odds of HCV were: having been previously tested for HIV and having fewer than 3 sexual partners in the previous 3 months.

### Regional differences in HIV and HCV seroprevalence

Using an unadjusted Fisher’s exact test, participants from the Coast were more likely to live with HIV (p < 0.001) and test seropositive for HCV (p < 0.001) (Table 1). In the multivariate analysis, partners from the Coast had 1.68 times higher odds of living with HIV (95% CI 1.13–2.51) (Table 2) and 1.72 times higher odds of being HCV antibody positive (CI: 0.84–3.74), although the HCV result was not significant (Table 3). After adjusting for sex and age, partners who had injected more than 5 years had 1.70 times (95% CI 1.34–2.61) higher odds of HIV on the Coast and 1.30 times higher odds in Nairobi (95% CI 1.12–1.50), and more than twice the odds of testing HCV seropositive in both regions (95% CI Coast = 1.61–6.01, 95% CI Nairobi = 1.69–3.24) with no evidence of interaction by region.

The majority of associations with HIV and HCV seroprevalence fell in the same direction for both regions, although there was modest evidence (p < 0.05) of interaction by region for several variables (Tables 2, 3). For HIV, older age (p_interaction_ = 0.004), being male (p_interaction_ = 0.03) being divorced (p_interaction_ = 0.04), and having given money for sex (p_interaction_ = 0.004) were stronger risk factors in Nairobi vs the Coast. Having been previously tested for HCV (p_interaction_ = 0.02), having used benzos in the past month (p_interaction_ < 0.001), and having 1–2 sexual partners in the past three months (ref = 0) (p_interaction_ = 0.002) were stronger risk factors for HIV in the Coast vs Nairobi.

For HCV, having been identified as an injecting partner (p_interaction_ = 0.04), having stable housing (p_interaction_ = 0.003), having experience physical violence (p_interaction_ = 0.005),having used heroin the past month (p_interaction_ < 0.001) and times injecting per month (p_interaction_ = 0.006) were both more strongly associated on the Coast vs Nairobi, while being partnered (p_interaction_ = 0.009), having > 2 sexual partners in the past three months (p_interaction_ = 0.01), receiving money for sex (p_interaction_ = 0.004), and being on methadone (p_interaction_ = 0.029) were more strongly associated with HCV in Nairobi vs Coast. Although the majority of partner characteristics did not show significant evidence for interaction with region in their association with HIV and HCV seroprevalence, sexual behaviors tended to have higher ORs for HIV in Nairobi while injecting behaviors tended to have higher ORs for HCV on the Coast (Tables 2, 3, Figs. 1, 2).

## Discussion

Overall HIV and HCV seroprevalence were high among sexual and injecting partners of PWID in Kenya, and we identified regional differences within Kenya in these prevalences and in the behaviors of sexual and injecting partners of HIV-positive PWID. The benefit of using APS to recruit both sexual and injecting partners of HIV positive PWID is that we were able to identify participants with elevated risk for HIV and HCV, often belonging to hard-to-reach key populations, a study that has not been conducted in Kenya and SSA. Importantly, our study also includes participants at elevated risk due to having sexual partners who live with HIV but who would not otherwise be identified in studies limited to key populations.

After adjusting for sex, age, and years injecting and accounting for clustering by recruitment site, HIV prevalence among sexual and injecting partners of PWID was more than threefold the national prevalence in Nairobi and more than fourfold on the Coast. HCV seroprevalence was at least tenfold higher in both regions. Consistent with prior studies [6, 13, 14], we observed a higher prevalence of HIV in Coastal Kenya, where partners had about 1.5 times the odds of living with HIV. Higher rates of risk-associated sexual behaviors may partially explain the higher seroprevalence of HIV in this region, although more research is needed to confirm this. While participants from the Coast were 1.72 time more likely to test seropositive for HCV compared to those from Nairobi, this difference was not significant after adjustment and is somewhat surprising given the higher prevalence of risk-associated injecting behaviors observed in Nairobi.

Participants from the Coast were more likely to be male, and male sex was associated with HCV in this study, although this trend is not consistently observed in other studies in SSA [6, 20]. Kenya has the greatest sex-disparity in IDU in Africa, with 93% of PWID being male [3, 27], suggesting that sex differences in HCV prevalence could be linked to difference in IDU behaviors by sex. This trend is reflected in our data, with males having 1.57 times higher odds of injecting drugs than females and reporting an average of two more years injecting compared to female participants, although the sex-difference also persisted among PWID in this study, with 16.5% of males who injected drugs, but only 7.3% of females who injected drugs, testing positive for HCV. We did not observe differences in the frequency of risk-associated injecting behaviors (needle or equipment sharing or injecting blood) by sex that could explain this trend. Sexual behaviors are unlikely to account for the sex-differences in HCV seroprevalence in this study because HCV is not readily transmitted through sex.

Participants recruited from the Coast were more likely to report sexual behaviors that have been associated with STI acquisition in other studies, and this may have contributed to the higher prevalence of HIV among participants recruited from the Coast [24, 28, 29]. With the exception of receiving money for sex, the sexual history variables that we looked at were associated with HIV seropositivity (although giving money for sex showed evidence of interaction by region and was only significantly associated in Nairobi and condom use was negatively associated, likely because of reverse causation). There was also a much higher prevalence of HIV among females. This trend has also been found in other studies of PWID in SSA [6, 20], although most of these studies have focused on PWIDs or people who engage in sex work, and there is limited data on those who engage in sex work and inject drugs, a population who we were able to reach using APS.

HCV is up to 4-times more infectious than HIV and is most often transmitted via transfusions or non-sterile injections [5]. Previous studies show that injecting behaviors are strongly associated with HCV. For example, Beckerleg et al. found that HIV prevalence was 3.8% among people using heroin without injecting but 61% among those who injected heroin [17]. Akiyama et al. found that more years injecting and more injections in the part month were associated with HCV on the Coast and in NairobI [6]. Therefore, we expected to see higher rates of IDU and/or risk-associated injecting practices in Coastal Kenya where HCV is more prevalent in our population. However, partners from Nairobi actually reported more risk-associated injecting behaviors, although the prevalence of IDU and the average years injecting was similar between both regions**.** This finding is surprising because an influx of drugs like heroin reached Costal Kenya before Nairobi [3]. The similarities in the prevelence of IDU and of years injecting between the two regions could support spread of heroin inland; however, we do not know the extent to which the regional trends we observed are specific to a population identified through APS. Higher rates of risk-associated injecting practices among participants recruited from Nairobi may also suggest that important interventions like NSP and/or education are less effective/less available or that PWID [30] face more barriers, such as hopelessness, to practicing safer injections in Nairobi compared to the Coast.

In our study, most partner characteristics showed similar associations with HIV and HCV seropositivity in the Coast and Nairobi and nearly all showed effects in the same direction. However, we did find that the association between HCV seropositivity and injecting history/behaviors like being enrolled as an injecting partner, injecting for 5 or more years, times injecting per month, and sharing equipment tended to be stronger on the Coast, although significant evidence of interaction by region was only observed for enrollment (p_interaction_ = 0.04) and times injecting (p_interaction_ = 0.006). Given the overall lower prevalence of injecting practices like needle/injection equipment sharing on the Coast, this disparity does not appear to be driven by differences in the behaviors of PWID in both regions. Instead, because there is a greater underlying prevalence of HCV on the Coast [6], participants in this region probably have a greater likelihood of being exposed to HCV even with fewer risk-associated injecting practices.

Our findings suggest that current interventions may be helping to increase the safety of IDU practices but highlight the need for continued and greater support for interventions and efforts to reach vulnerable populations. In both Nairobi and the Coast, almost 70% of participants who injected drugs reported having done so for less than six years. We would, therefore, expect to see high rates of risk-associated injecting practices in our study, as other studies suggest that new injectors (injecting for < 6 years) are more likely to participate in risky injection practices such as sharing needles/injecting equipment, and less likely to participate in HIV prevention programs like NSP and drug treatment options [31, 32]. Given that the risk of testing HIV positive or HCV antibody positive increases with the number of years injecting and with age [6, 33–36], the prevalence of HIV and HCV may continue to rise as this population ages; however, we would expect the incidence to attenuate over time [13].

Overall, PWID in our study were slightly less likely to report sharing syringes than PWID in similar recent studies and drastically less likely to report sharing than participants from studies conducted prior to the introduction of NSP [6, 7, 24]. While some of this disparity may reflect differences in how participants were sampled, this difference also suggests that services like NSP, methadone, and/or education are reaching the sexual and injecting partners of HIV positive PWID and may be helping them to follow safer injecting practices. However, the higher rates of risk-associated injecting practices in Nairobi raise concern and need to be addressed.

Strengths of this study include that this is the only multi-site studies among sexual/injection partners of PWID to document HIV and HCV antibody prevalence in Kenya and SSA, therefore results from this study could inform population-specific prevention interventions. Limitations of this study include that we reported HCV prevalence based on the results from rapid antibody test which might be an imperfect measure of active HCV infection [37]. We have also focused only on the sexual and injecting partners of HIV positive PWID and, by identifying participants through APS, we are not able to assess the prevalence of the characteristics we looked at in the general population. The results from this study are likely to have been affected by social desirability bias resulting from the sensitive nature of information collected from the study participants e.g. sexual and injection history. The sampling method used was convenience sampling which is associated with selection bias and therefore results from this study may not be generalization to other populations. Clustering effect by site was included in the association model but cannot capture all sources of clustering related to the use of APS to recruit participants. This study is a descriptive study and none of the associations described can be assumed to be causative. However, our results show important trends, such as the higher prevalence of HIV and HCV in the Coast but higher prevalence of risk-associated injecting behaviors in Nairobi.

## Conclusion

Studies show that new injectors such as those represented in our study, are at elevated risk of contracting HIV and/or HCV. Providing resources and education will be crucial to reduce exposure and to maintain the encouragingly lower needle and equipment sharing that we observed compared to other studies. Despite minimal difference in significant risk factors between the two regions, reducing transmissions is likely to be especially challenging on the Coast, where interventions must combat an underlying higher prevalence of HIV and HCV. Interventions should also bear in mind that females may be at higher risk of HIV and males at higher risk of HCV in this population, and future studies should investigate the cause of this disparity.

## Supplementary Information

**Additional file 1: Table S1.** Missingness among analyzed variables for all enrolled and in final dataset used in analysis. **Table S2.** Matches Table 1, but showing both categories for binary variables.

## Data Availability

The datasets generated and/or analyzed during the current study are not publicly available because the local ethics review committee require oversight of use of research data but are available from the corresponding author on reasonable request.

## Abbreviations

APS: Assisted partner services
HCV: Hepatitis C virus
IDU: Injecting drug use
IQR: Interquartile range
NASCOP: National AIDS and STI Control Programme
NSP: Needle syringe program
PWID: Persons who inject drugs
SSA: Sub-Saharan Africa
