# Supplementary material for: Characteristics associated with HIV and hepatitis C seroprevalence among sexual and injecting partners of HIV positive persons who inject drugs in Nairobi and coastal Kenya
Source: BMC Infect Dis. 2022 Jan 21;22:73. doi: 10.1186/s12879-022-07036-8 (PMC8780315; doi:10.1186/s12879-022-07036-8)
Supplement: Supplementary file 1 — Additional file 1: Table S1. Missingness among analyzed variables for all enrolled and in final dataset used in analysis. Table S2. Matches Table 1, but showing both categories for binary variables. [file 12879_2022_7036_MOESM1_ESM.pdf]

	All enrolled (N=2,462)		Final dataset (N=2,386)	
**variable**	**N missing**	**% missing**	**N missing**	**% missing**
times injecting per month*	270	11	215	9
shared needles (past month)*	270	11	215	9
shared equipment (past month)*	270	11	215	9
injected blood (past month)*	270	11	215	9
on methadone now*	270	11	215	9
years injecting	53	2.2	0	0
used heroin (past month)	47	1.9	0	0
used benzos (past month)	47	1.9	0	0
used cocaine (past month)	47	1.9	0	0
used a condom when last had sex	34	1.4	28	1.2
partner type	28	1.1	28	1.2
Positive HCV test	17	0.69	0	0
Positive HIV test	11	0.45	0	0
sex_partners_3mo_group	7	0.28	3	0.13
sex_partners_3mo_group.1	7	0.28	3	0.13
Received money for sex (ever)	7	0.28	3	0.13
gave money for sex (ever)	7	0.28	3	0.13
had sex with someone knew to be HIV positive (ever)	7	0.28	3	0.13
sex	5	0.2	0	0
age	5	0.2	0	0
marital status	4	0.16	0	0
have stable housing	4	0.16	0	0
previously tested for HIV	4	0.16	0	0
previously tested for HCV	4	0.16	0	0
experienced physical violence (past year)	4	0.16	0	0
site enrolled	0	0	0	0
identified by multiple partners	0	0	0	0
Previously tested positive for HIV	0	0	0	0
Previously tested for HCV	0	0	0	0
used alcohol (past month)	0	0	0	0
region recruited	0	0	0	0

*These variables were analyzed only among PWID, so missingness includes people who do not inject drugs by design

Supplemental table 1. Missingness among analyzed variables for all enrolled and in final dataset used in analysis.

	Coast (N=1026)	Nairobi (N=1360)	Total (N=2386)	p value
**site**				<0.001
Githurai	0 (0.0%)	375 (27.6%)	375 (15.7%)	
Kilifi-MEWA	79 (7.7%)	0 (0.0%)	79 (3.3%)	
Malind-Omari	445 (43.4%)	0 (0.0%)	445 (18.7%)	
Mtwapa-MEWA	194 (18.9%)	0 (0.0%)	194 (8.1%)	
Ngara	0 (0.0%)	593 (43.6%)	593 (24.9%)	
Pangani	0 (0.0%)	392 (28.8%)	392 (16.4%)	
Reachout	308 (30.0%)	0 (0.0%)	308 (12.9%)	
SOCIODEMOGRAPHIC CHARACTERISTICS				
**partnertype**				<0.001
Both sexual & Injecting	236 (23.4%)	211 (15.6%)	447 (19.0%)	
Injecting	602 (59.7%)	928 (68.7%)	1530 (64.9%)	
Sexual	170 (16.9%)	211 (15.6%)	381 (16.2%)	
**number of times named**				<0.001
once	736 (71.7%)	1168 (85.9%)	1904 (79.8%)	
more_than_once	290 (28.3%)	192 (14.1%)	482 (20.2%)	
**sex**				<0.001
Female	230 (22.4%)	521 (38.3%)	751 (31.5%)	
Male	796 (77.6%)	839 (61.7%)	1635 (68.5%)	
**age**				<0.001
Mean (SD)	35.3 (7.6)	31.8 (8.0)	33.3 (8.0)	
Range	19.0 - 60.0	18.0 - 74.0	18.0 - 74.0	
Median (IQR)	35.0 (30.0, 40.8)	31.0 (25.0, 37.0)	33.0 (27.0, 39.0)	
**marital_status**				<0.001
Single	319 (31.1%)	682 (50.1%)	1001 (42.0%)	
Divorced	268 (26.1%)	276 (20.3%)	544 (22.8%)	
Partnered	93 (9.1%)	53 (3.9%)	146 (6.1%)	
Married_or_widowed	346 (33.7%)	349 (25.7%)	695 (29.1%)	
**have stable housing**				0.0011
No	111 (10.8%)	210 (15.4%)	321 (13.5%)	
Yes	915 (89.2%)	1150 (84.6%)	2065 (86.5%)	
**experienced physical violence (past year)**				<0.001
No	574 (55.9%)	944 (69.4%)	1518 (63.6%)	
Yes	452 (44.1%)	416 (30.6%)	868 (36.4%)	
HIV/HCV HISTORY AND TEST RESULTS				
**previously tested for HIV**				<0.001
No	98 (9.6%)	32 (2.4%)	130 (5.4%)	
Yes	928 (90.4%)	1328 (97.6%)	2256 (94.6%)	
**previously tested positive for HIV**				<0.001
No	814 (79.3%)	1166 (85.7%)	1980 (83.0%)	
Yes	212 (20.7%)	194 (14.3%)	406 (17.0%)	
**previously tested for HCV**				<0.001
No	898 (87.5%)	986 (72.5%)	1884 (79.0%)	
Yes	128 (12.5%)	374 (27.5%)	502 (21.0%)	
**previously tested seropositive for HCV**				0.91
No	991 (96.6%)	1315 (96.7%)	2306 (96.6%)	
Yes	35 (3.4%)	45 (3.3%)	80 (3.4%)	
**HIV positive test**				<0.001
FALSE	787 (76.7%)	1130 (83.1%)	1917 (80.3%)	
TRUE	239 (23.3%)	230 (16.9%)	469 (19.7%)	
**HCV seropositive test**				<0.001
FALSE	847 (82.6%)	1242 (91.3%)	2089 (87.6%)	
TRUE	179 (17.4%)	118 (8.7%)	297 (12.4%)	
SEXUAL HISTORY				

**number of sexual partners (past 3 months)**				<0.001
0	330 (32.2%)	811 (59.7%)	1141 (47.9%)	
1-2	483 (47.1%)	390 (28.7%)	873 (36.6%)	
>2	212 (20.7%)	157 (11.6%)	369 (15.5%)	
**received money for sex (ever)**				<0.001
No	572 (55.8%)	877 (64.6%)	1449 (60.8%)	
Yes	453 (44.2%)	481 (35.4%)	934 (39.2%)	
**gave money for sex (ever)**				<0.001
No	418 (40.8%)	912 (67.2%)	1330 (55.8%)	
Yes	607 (59.2%)	446 (32.8%)	1053 (44.2%)	
**had sex with someone knew to be HIV positive (ever)**				<0.001
No	799 (78.0%)	1211 (89.2%)	2010 (84.3%)	
Yes	226 (22.0%)	147 (10.8%)	373 (15.7%)	
**used a condom when last had sex**				0.11
No	569 (56.4%)	716 (53.1%)	1285 (54.5%)	
Yes	440 (43.6%)	633 (46.9%)	1073 (45.5%)	
DRUG USE				
**used heroin (past month)**				<0.001
No	149 (14.5%)	74 (5.4%)	223 (9.3%)	
Yes	877 (85.5%)	1286 (94.6%)	2163 (90.7%)	
**used benzos (past month)**				0.35
No	856 (83.4%)	1114 (81.9%)	1970 (82.6%)	
Yes	170 (16.6%)	246 (18.1%)	416 (17.4%)	
**used cocaine (past month)**				<0.001
No	913 (89.0%)	1291 (94.9%)	2204 (92.4%)	
Yes	113 (11.0%)	69 (5.1%)	182 (7.6%)	
**used alcohol (past month)**				0.54
No	679 (66.2%)	917 (67.4%)	1596 (66.9%)	
Yes	347 (33.8%)	443 (32.6%)	790 (33.1%)	
INJECTION DRUG USE BEHAVIORS				
**years injecting**				0.085
0 (don't inject)	95 (9.3%)	121 (8.9%)	216 (9.1%)	
<5	540 (52.6%)	776 (57.1%)	1316 (55.2%)	
≥5	391 (38.1%)	463 (34.0%)	854 (35.8%)	
**times injecting per month**				0.49
Mean (SD)	70.1 (51.5)	72.4 (94.0)	71.4 (78.6)	
Range	0.0 - 360.0	0.0 - 3000.0	0.0 - 3000.0	
Median (Q1, Q3)	60.0 (30.0, 90.0)	60.0 (56.0, 90.0)	60.0 (30.0, 90.0)	
**shared needles (past month)**				<0.001
No	909 (97.6%)	1168 (94.2%)	2077 (95.7%)	
Yes	22 (2.4%)	72 (5.8%)	94 (4.3%)	
**shared equipment (past month)**				<0.001
No	904 (97.1%)	1053 (84.9%)	1957 (90.1%)	
Yes	27 (2.9%)	187 (15.1%)	214 (9.9%)	
**injected blood (past month)**				1
No	917 (98.5%)	1222 (98.5%)	2139 (98.5%)	
Yes	14 (1.5%)	18 (1.5%)	32 (1.5%)	
**on methadone**				<0.001
No	600 (64.4%)	1094 (88.2%)	1694 (78.0%)	
Yes	331 (35.6%)	146 (11.8%)	477 (22.0%)	
**stopped_methadone now**				<0.001
No	331 (74.4%)	146 (57.3%)	477 (68.1%)	
Yes	114 (25.6%)	109 (42.7%)	223 (31.9%)	

Supplemental Table 2. Matches Table 1 from, but showing both categories for binary variables.
